# P-917. Don't Underestimate Your Impact! Pharmacist and Physician Validation of Adult Antimicrobial Stewardship Intervention Impact Scoring

**DOI:** 10.1093/ofid/ofaf695.1123

**Published:** 2026-01-11

**Authors:** Amanda Lefemine, Erin Pollock, Michael S Boger, Julie E Williamson, Mahlet Gebrekidan, Lisa Davidson

**Affiliations:** Advocate Health: Atrium Health Antimicrobial Support Network, Charlotte, NC; Advocate Health: Atrium Health Antimicrobial Support Network, Charlotte, NC; Advocate Health: Atrium Health Antimicrobial Support Network, Charlotte, NC; Advocate Health: Atrium Health Antimicrobial Support Network, Charlotte, NC; Advocate Health: Atrium Health Antimicrobial Support Network, Charlotte, NC

## Abstract

**Background:**

Antimicrobial stewardship programs (ASPs) are essential for safe, effective, and optimal antimicrobial use. Though documented ASP interventions are frequently measured quantitatively by programs, an objective, reproducible method to measure the impact of these interventions on patient safety and antimicrobial use metrics has remained challenging.

**Figure 1.**
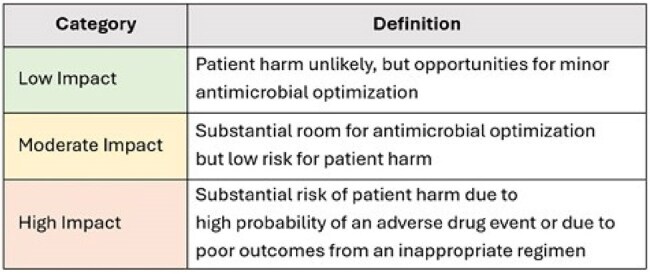
Impact Category Definitions

**Figure 2.**
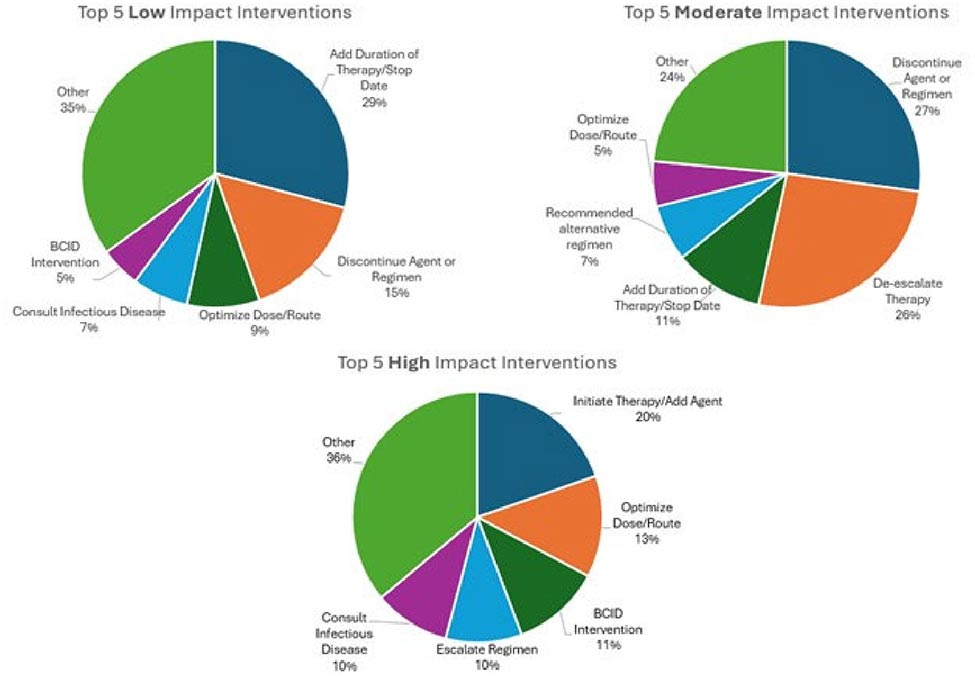
Top 5 Low, Moderate, and High Impact Intervention Types

Charts represent intervention breakdown based on pharmacist scoring. BCID (Blood Culture Identification) interventions are those made based on rapid diagnostic test results of positive blood cultures, and these are typically linked to other intervention types.

**Methods:**

Our ASP adapted an impact ranking system from a previously published scoring tool which categorizes each intervention type as low, moderate, or high impact based on risk of patient harm and degree of antimicrobial optimization (Figure 1). This is a retrospective review of interventions from December 2024 through February 2025 across a large healthcare system including 11 hospitals of varying acuity and size. Each intervention was reviewed by both an infectious diseases (ID) physician and pharmacist. The primary aim of this study is to characterize the impact of different types of ASP interventions to optimize stewardship opportunities.

**Figure 3.**
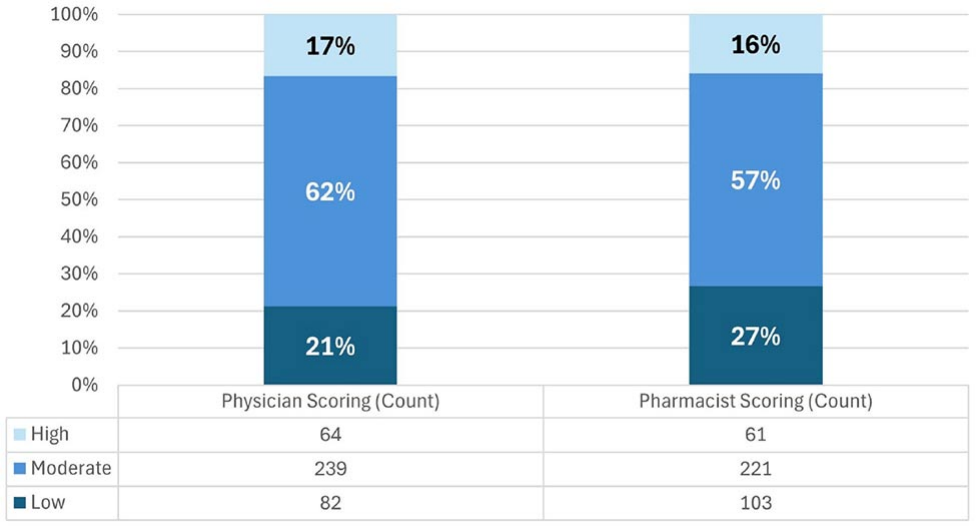
Physician and Pharmacist Impact Scoring

**Results:**

A sample of 385 interventions are included in this analysis of 7,402 interventions completed during the study period. The most common impact level was moderate (57%) followed by low (27%). High impact interventions were most often related to initiating therapy (20%) whereas low and moderate interventions were more commonly related to durations of therapy, discontinuations, and de-escalations (Figure 2). The impact scoring level agreement between physician and pharmacist was 82% (Figure 3). The most common interventions with discordance were adding duration of therapy, ordering labs/tests for monitoring, and discontinuing agent/regimen, with physicians scoring the interventions as higher impact most often.

**Conclusion:**

Our analysis demonstrated that ASP pharmacists frequently perform high and moderate impact interventions that prevent what otherwise may have led to undesirable outcomes. There is a general agreement between ID pharmacists and physicians on the impact of these interventions. Understanding the impact level of different intervention types may assist in efficient and effective use of ASP resources.

**Disclosures:**

All Authors: No reported disclosures

